# Association between multiple sclerosis, cancer risk, and immunosuppressant treatment: a cohort study

**DOI:** 10.1186/s12883-017-0932-0

**Published:** 2017-08-08

**Authors:** Paolo Ragonese, Paolo Aridon, Giulia Vazzoler, Maria Antonietta Mazzola, Vincenzina Lo Re, Marianna Lo Re, Sabrina Realmuto, Simona Alessi, Marco D’Amelio, Giovanni Savettieri, Giuseppe Salemi

**Affiliations:** Dipartimento di Biomedicina Sperimentale e Neuroscienze Cliniche (Department of Experimental Biomedicine and Clinical Neurosciences), Università degli Studi di Palermo, Via G. La Loggia, 1, 90129 Palermo, Italy

**Keywords:** Multiple sclerosis, Cancer, Cohort study, Immunosuppressant, Treatment

## Abstract

**Background:**

The association between multiple sclerosis (MS) and cancer has long been investigated with conflicting results. Several reports suggest an increased cancer risk among MS patients treated with immunosuppressant (IS) drugs.

**Methods:**

We performed a cohort study including MS patients recruited at the Neurological Department of the University of Palermo. Mean follow-up period was ten years for the whole cohort. We calculated cancer incidence among patients treated with IS. Incidence rates were compared in the cohort by calculating the relative risk according to length and dose of exposure to IS. Cancer incidence among MS patients was compared to cancer incidence in the general population of Sicily in similar age groups.

**Results:**

On an overall cohort of 531 MS patients (346 women and 185 men) exposed to IS, we estimated a crude incidence rate for cancer of 2.26% (2.02% in women, 2.7% in men). Cancer risk was higher compared to rates observed among an equal number of patients not exposed to IS, and to the risk in the general population in Sicily at similar age groups (adjusted HR: 11.05; CI 1.67–73.3; *p* = 0.013).

**Conclusion:**

The present study showed a higher cancer risk in MS patients associated only to previous IS exposure. Studies on long-term outcomes are essential to evaluate the possibility that treatment options that need to be considered for a long time-period may modify risk for life threatening diseases.

## Background

The pathologic mechanisms of Multiple sclerosis (MS) are mediated by the immune system and by neurodegenerative processes causing both myelin sheath and axonal damage. MS represents one of the major causes of neurological disability, especially in young people [1]. First line treatments for MS until recently, were basically represented by immune modulating drugs like beta interferon or glatiramer acetate, while in patients with more aggressive disease courses or who did not respond to first line treatments, immune suppressors (IS) had been used [2]. For these reasons, azathioprine (AZA) was still in use, even after the introduction of disease modifying treatments (DMT). Mitoxantrone (MX) and cyclophosphamide (CP) were mostly chosen as second line treatments or as induction therapy in patients with a more aggressive disease. Their use declined significantly after the introduction of new therapies characterized by a specific mechanism of action for the treatment of MS, like monoclonal antibodies (natalizumab was approved in Italy in 2006) and fingolimod (introduced in Italy 2011) though these drugs still remain a therapeutic alternative in selected cases. The safety profile of immune treatments for MS [3–7], as well as the relationship between the disease and cancer, has been widely explored but results have been yet not conclusive [8–14]. These inconsistencies are due to methodological differences in study design, by the difficulty in selecting an appropriate reference group for comparisons, and length of follow-up, which is of particular relevance in clinical trials exploring the safety of drugs. Several question have not been yet well addressed. Do generally IS used for MS increase the risk for cancer or is there a particular IS which is mainly associated with increased cancer risk? Is there a difference between cancer risk among MS patients treated with IS and those who were not exposed to IS, compared to people not affected by MS (general population)? In order to contribute to assess these issues we performed a cohort study including patients affected by MS exposed to IS, and an equal number of MS patients not exposed to IS to determine cancer risk. The present study was also aimed to compare cancer incidence among people affected by MS to that in the general population in the same geographic area and in the same period.

## Methods

### Cohort design

During the study period (1994, to January 31st, 2011) we identified at our Neurologic Department a cohort of 531 MS patients affected by MS (346 women and 185 men) who took at least one of the three IS (AZA, MX, and CP) independently if they had been treated with interferon or glatiramer. An equal number of patients (531) with the same sex distribution but not exposed to IS was matched as reference group. To assess the above-mentioned questions in the cohort of MS patients we considered two kind of comparison groups and hence, two different approaches. A first group consisted as indicated in an equal number of individuals affected by MS consecutively selected and followed in the same time interval, among patients followed at the same Neurological Department who had never been treated with IS. We also compared cancer incidence among individuals affected by MS exposed or not to IS, with that in the Sicilian general population, considering similar age groups and calculating the standardized incidence ratio (SIR) for cancer in the two cohorts.

### Treatments

MX and CP were used in our Department after other treatments had failed to reduce relapse frequency or disability worsening in patients affected by MS. Before starting MX, patients underwent a screening for cardiovascular diseases. Treatment administration was made at a monthly dose of 8 mg/m^2^ for the first three months, and at a dose of 12 mg/m^2^ every three months up to a cumulative dose of 140 mg/m^2^. We used this regimen from 1994 until 2000. Afterward to limit cumulative long-term toxicity, we modified the cumulative dose limiting it to a maximum of 90 mg/m^2^. We limited the cumulative dose of MX because of the already known possible heart toxicity. For the same reason, we used to perform a tight monitoring of heart function by trans-thoracic ecocardiography. Treatment was stopped before reaching the planned final dose if signs of impaired heart function, such as confirmed left ventricular ejection fraction (LVEF) reduction to less than 50%, or a confirmed reduction of more than 10% with respect to the first examination, were observed. Reduction or termination of infusions due to adverse events was at the physician’s judgment. Azathioprine was targeted to reach a maximum dose of 3 mg/Kg of body weight per day if tolerated. Decrease of IS dosages were done in the presence of severe leucopoenia, red blood cell or platelet reduction, severe infections or other clinically relevant serological markers modifications. CP was administered at a monthly dose of 500 mg/m^2^ during the first year of treatment; it was eventually prolonged during the second year with courses repeated every two months. We included in the whole cohort MS patients who were treated with the three IS considered in the same lag-time, obtaining a cohort with a comparable follow-up. The second group of MS patients (represented as above indicated by 531 individuals) were treated with other drugs available at that time (beta interferon or glatiramer).

### Statistical analyses

Calculations of outcome measures were performed considering January 31st, 2011 as the end of follow-up while the observation was started in 1994 at the time of inclusion of the first patient for MX treatment. Statistical analyses were performed by calculating univariate relative risks (RR) for cancer comparing its incidence in MS patients exposed to IS with that in those not exposed to IS. Cox proportional models were built to calculate adjusted hazard ratios (HR) considering the follow-up period as the time variable. In these models, age at MS onset, and length of the follow-up expressed in years, were calculated as continuous variables. A separate Cox model was built including also age at treatment initiation (IS or other therapies) as a covariate. We performed also a second analysis by comparing cancer incidence rates in our cohort of MS patients, with that of individuals from the Sicilian general population of the same age groups. By means of this kind of comparison, we calculated the possible difference existing in the probability to develop cancers according to the condition of being affected or not by MS itself and not only on being exposed to therapy. Since MS cohort was represented by individuals in the age classes between 18 and 54 years of age, we made comparisons by including as a reference, cancer incidence in the Sicilian population at the same age groups. These data were freely available from the Italian National tumor registry (http://www.tumori.net/). In these second analyses, we calculated SIR. All the analyses were two-sided with alpha level set at 0.05; 95% confidence intervals (CI) were also calculated.

The local ethical committee of our University Hospital approved the study and each patient gave the consent for treatment and inclusion in our clinical database.

## Results

We included 531 MS patients from our database (346 women and 185 men), who had been treated with IS. 531 MS individuals who had never been exposed to IS were included for comparisons. Table 1 summarizes characteristics of the two groups of MS patients in the cohort. The two groups were not significantly different with regard to age, disease duration and cumulative length of the follow-up. Mean follow up was 9 years for patients treated with IS, and 10 years in for the others. As expected, patients treated with different kind of IS have different period of treatment exposure; AZA treatment was usually stopped after 5 years of therapy, while for CP and MX patients usually reached the planned total cumulative dose within two tears of therapy. Details about types of tumors in men and women are reported in Table 2. Gut cancers (colon or rectal cancer), female breast cancer and leukemia represented the most common type of cancer. Cancer distribution according to length of exposure and the type of IS are reported in Table 2. This variable was dichotomized according to the median time of treatment duration for each specific drug (below or above the mean). In the present cohort, we observed no cancers in those people exposed to CP alone. Table 3 reports relative risks and hazard ratios for the association between MS and the exposure to IS. At univariate analyses, we observed a significant association between exposure to IS and cancer in MS patients. Individuals treated with AZA had a four-fold increased risk for cancer, compared to those not exposed (median time of treatment 5 years). Among MS patients who were treated with MTX (median exposure one year), cancer risk was four times higher. Cox proportional hazard models revealed an even higher risk for cancers associated to IS exposure; HR was 11.05 (95% CI 1.67–73.30; *p* = 0.01) in the most conservative model. Age at onset of MS appeared to be independently associated to cancer risk (HR 1.05; CI 1.01–1.10; *p* = 0.03). As indicated in Table 4, SIR was four times higher in MS individuals exposed to IS compared to the age matched general population. On the contrary there was no a different cancer incidence in people affected by MS who did not undergo to IS therapy, showing significant lower cancer rates (SIR 0.97; CI 0.96–0.98).

**Table 1:** Demographic and clinical characteristics of the MS cohort included in the study

Variable	Exposed to IS	Others
Sex (W/M)	531 (336/195)	531 (359/172)
Mean age at onset (years +/− SD)	29 (10)	30 (10)
Mean age at treatment start (years +/− SD)	29.4 (9.9)	40 (13.5)
Mean disease duration (SD)	19 (9)	20 (10)
Mean Follow-up (years +/− SD) for the whole cohort	9 (5)	10 (6)
Mean Follow-up (years +/− SD) for the MX group	8 (5)	_
Mean Follow-up (years +/− SD) for the CP group	5 (5)	_
Mean Follow-up (years +/− SD) for the AZA group	10 (6)	_
Mean treatment duration for the MX group (years)	1	_
Mean treatment duration for the CP group (years)	1	_
Mean treatment duration for the AZA group (years)	4	_

**Table 2:** Number and type of cancer by IS drug and by exposure duration

Drug	Exposure duration (years)	No. of patients	N. and site of cancer	p*
Azathioprine	< 4	169	3 (gut ^a^, brain, ovary)	
	≥4	177	4 (3 breast, 1 gut^a^)	0,75
Mitoxantrone	<1	74	1 (pancreas)	
	≥1	188	5 (3 leukemia, 1 lung, 1 gut)	0,5

* two sided Chi square analysis comparing the frequency among MS patients exposed for the longer period, compared to those exposed for a shorter period

^a^Gut cancers include colon and rectal cancers

**Table 3:** Relative risks and HR calculation by investigated variables

Variable	RR (CI)°	HR	p
Model A			
Sex (men/women)	0.78 (0.28–2.22)	1.24 (0.43–3.56)	0.69
Age at MS onset^a^	1.001 (0.97–1.03)	1.05 (1.01–1.10)	0.03
Follow-up length^a^	1.03 (0.98–1.08)	_	_
IS exposure (yes vs. no)^b^	4.01 (1.14–14.1)	11.05 (1.67–73.3)	0.013
Model B			
Sex		1.32 (0.46–3.78)	0.6
Age at treatment^a^		1.07 (1.02–1.12)	0.01
Disease duration^a^		2.36 (0.71–7.84)	0.2
IS exposure (yes vs. no)^b^		35.05 (3.12–403.31)	0.01

^a^Age at onset and length of follow-up were dichotomous variables according to the median of the distribution in the whole cohort in univariate analyses, while they were calculated as continuous variables in Cox models

^b^Model B was built including age at treatment initiation (IS or other therapies) and disease duration as covariates

**Table 4:** SIR and 95% confidence intervals calculations for MS patients exposed or not to IS drugs. Comparison were made with respect to the Sicilian general population in the correspondent age classes

Age	Exposed to IS			Not exposed to IS		
	No.	Expected	Observed	No.	Expected	Observed
15–19	1	0,00035	0	2	0,0007	0
20–24	2	0,00100	0	9	0,0050	0
25–29	17	0,01200	0	28	0,0210	0
30–34	35	0,03600	0	41	0,0420	0
35–39	71	0,10,000	1	61	0,0900	0
40–44	66	0,16,000	2	78	0,1900	0
45–49	87	0,30,000	1	78	0,2700	1
50–54	90	0,48,000	3	59	0,3160	0
55–59	62	0,47,000	1	56	0,4280	0
60–64	48	0,51,000	2	52	0,5500	0
65–69	35	0,52,000	0	34	0,5100	1
70–74	13	0,24,000	3	17	0,3100	0
75–79	4	0,08600	0	7	0,1500	0
80–84	0	0,00000	0	6	0,1400	1
85+	0	0,00000	0	3	0,0650	0
Total	531	2,91,535	13	531	3,0877	3
SIR	Tot		4,12			0,97

## Discussion

The present study did not show an association between being affected by MS and cancer. The study revealed, a direct strong association between IS exposure among individuals affected by MS and cancer. Risk for cancer observed in individuals with MS exposed to IS seems to be related to the duration of exposure and to the cumulative dose, not to a specific IS. Our study has some limits derived from the relatively small number of cancers identified in the cohort, despite the fact that a large cohort of MS patients was included and the analyses were performed on the basis of a long follow-up period. Our aim was to investigate if the risk for cancer could be related to the disease itself or to the exposure to treatments. Therefore we chose to compare MS patients exposed to IS to MS individuals who had never been treated with IS, and to a second cohort of individuals not affected by MS. For the same reason we compared cancer incidence in MS individuals to that of the Sicilian general population. In our cohort, we did not observe any cancer among individuals who were exposed to cyclophosphamide only, similarly to other study [15]; it is to note however, that in our cohort, the group of patients treated with CP was the smallest and the follow-up was not as long as for the other drugs investigated. Cancer risk in the present study appears to be similar in individuals treated with MTX or AZA. These results confirm previous studies indicating a higher cancer risk related to MTX exposure, represented not only by leukemia [7, 10]. We also observed similar results for azathioprine; breast cancer was the most frequently observed in this group of patients. The results we observed need several comments. A positive association between MS and cancer has been proposed several times, suggesting that chronic inflammation could be the mechanism underneath this relationship [16]. More recent observations, anyway, do not support this association anymore, although conflicting results were reported for the association between MS and specific kind of cancers in several studies [14, 17]. A study investigating the association between autoimmunity and cancer risk [17], showed an inverse association between MS and intestinal cancers. Other studies did not confirm this association reporting on the contrary a global reduction of cancer risk and an association with other kind of tumors [8, 17, 18].

We found that MS does not appear to be associated to cancer risk by itself. Although we did not observe relevant differences between men and women for cancer risk, these results were not plotted because further stratification would have led to small numbers and very broad confidence intervals. Another limitation of this study derives from the fact that due to the study design, it was not possible to investigate the possible confounding effect of environmental risk factors for cancer like smoking habit. It would be in fact not feasible to obtain this information for referent individuals from the general population. Another important consideration derives from the fact that all the analyses performed with the two different approaches we used, lead to the same observation in our cohort. Comparing MS patients’ risk for cancer with the one in the general population with similar ages reduced also the possibility of a possible selection bias deriving from age at patients’ selection. It is also important to underline that length of follow-up is crucial in this kind of studies. Carcinogenesis may occur in fact with a long latency. Studies with shorter follow-up may not be able to identify an association also because in the age groups represented by MS patients, cancer incidence depends on the anatomical and histological type of the tumor. All of the three drugs considered in this study act suppressing specific lymphocyte subpopulation but also by permanently modifying DNA expression of oncogenes; all of them activate pathways that determine an increased risk for the development of cancers [19]. For instance, suppression of natural killer cells has been associated for instance to an increased risk for leukemia and for skin cancers in several studies although not focused to MS patients [20]. This is not of course the only explanation for the association we observed. Patients with chronic diseases like MS are frequently exposed to several drugs whose interaction may contribute to alter immune function leading to increased cancer susceptibility. However, there is no information about how the temporal interaction between drugs may further modify the risk. We did not observe anyway in our cohort a higher risk among those patients who were exposed to more treatments. Another explanation would consider the possibility that MS patients, similarly to what has been hypothesized in other immune mediated diseases, may have a higher intrinsic susceptibility to cancer if exposed to immune modifying drugs [21]. This hypothesis is not supported by our findings showing that MS individuals who have been treated with drugs different from IS, like beta interferon or glatiramer, did not show risk of developing cancer differently from that observed in the general population [22]. Another important methodological aspect needs to be highlighted. In our cohort of patients, we observed a higher age at treatment initiation among those people who were never treated with IS therapy. This should not appear surprising considering that immune suppression was the only available therapy for more aggressive cases until the availability of natalizumab. For this reason, in patients with a less aggressive disease course, there could be a longer latency from MS onset to treatment start particularly if we consider the possible effect on this phenomenon of the diagnostic criteria used at that time and also, for a possible different disposition to treat patients with a disease course considered less aggressive. Nevertheless, as shown in Table 3, the Cox model that included also age at treatment start (IS or other drugs) as a covariate, led an even higher HR for the association between IS and cancer risk. The observation that we did not show an association between the exposures to drugs like beta interferon or glatiramer, and cancer risk, is of particular interest. The necessity to balance between the need for a high impact drug with a possible long lasting effect on MS disease curse, and the consequent acceptance of reasonable risks in terms of safety, is in fact one of the most challenging aspects for neurologists taking care of people with MS. New drugs recently introduced or those that are going to be released for MS treatment act on specific pathways of immune system. These new mechanisms of action lack of the knowledge about long term effects. This study in our opinion strengthen the need to implement surveillance programs to improve our understanding of long term side effects and safety profile of drugs already in use and of new drugs which are going to be approved to treat MS. This would be particularly helpful for not common events that may occur after a long latency from the exposure.

## Conclusion

Our study showed a higher cancer risk in MS patients associated to previous IS exposure but not the disease itself. Even though not all the drugs we considered were associated to increased cancer risk, the present study indicate the need for careful selection of MS patients requiring therapies whose efficacies are balanced by potential harmful effects, and a stringent follow-up of those patients treated with IS. Despite the increasing amount of therapeutic possibilities to cure MS, patients still exist, who do not respond to available drugs and experience an unfavorable disease course, rapidly accumulating disability, or patients who have lack of tolerance and need therefore a therapeutic alternative. In all of these patients, a strict long-term follow-up must be planned to avoid life threatening conditions.

## Abbreviations

AZA: Azathioprine
CI: Confidence intervals
CP: Cyclophosphamide
DMT: Disease modifying treatments
HR: Hazard ratio
IS: Immunosuppressant drugs
LVEF: Left ventricular ejection fraction
MS: Multiple sclerosis
MX: Mitoxantrone
RR: Relative risk
SIR: Standardized incidence ratio
